# Characterization of Novel Cement-Based Carboxymethyl Chitosan/Amorphous Calcium Phosphate

**DOI:** 10.1055/s-0041-1739449

**Published:** 2022-01-11

**Authors:** Aditya Wisnu Putranto, Endang Suprastiwi, Ratna Meidyawati, Harry Agusnar

**Affiliations:** 1 Department of Conservative Dentistry, Faculty of Dentistry, Universitas Indonesia, Jakarta, Indonesia; 2 Department of Chemistry, Faculty of Mathematics and Natural Science, Universitas Sumatera Utara, Medan, Indonesia

**Keywords:** gypsum, CMC/ACP, carboxymethyl chitosan, amorphous calcium phosphate, dentin remineralization, Fourier-transform infrared spectroscopy, X-ray diffraction, scanning electron microscopy, setting time

## Abstract

**Objective** This study aimed to analyze, evaluate, and characterize novel cement-based carboxymethyl chitosan/amorphous calcium phosphate (CMC/ACP).

**Materials and Methods** The three cement groups studied were gypsum (Gyp), and CMC/ACP—gypsum cement-based 5% (5% CAG) and 10% (10% CAG). The groups were characterized using Fourier-transform infrared spectroscopy (FTIR), X-ray diffraction (XRD), setting time, and scanning electron microscopy (SEM) data. The characterization results were analyzed qualitatively, but the data for setting time were analyzed using SPSS ( *p* < 0.05).

**Statistical Analysis** Data were statistically analyzed. One-way analysis of variance was used to compare numerical (parametric) data between more than two separate groups followed by post hoc Tukey.

**Results** FTIR showed phosphate groups indicate the presence of calcium phosphate in the form of amorphous (ACP) in the CMC/ACP, CMC/ACP post-milled powder, and CMC/ACP cement-based (5% CAG and 10% CAG). XRD showed no difference in the diffraction spectra among the Gyp, 5% CAG, and 10% CAG groups. SEM images revealed that the CMC/ACP cement-based groups (5% CAG and 10% CAG) showed CMC/ACP cluster filled with hollow spaces between the gypsum crystals and aggregations surrounding the gypsum crystals. The CMC/ACP showed envelopes and attached to the crystalline structures of the gypsum. Setting times of 5% CAG and 10% CAG showed significant differences compared with Gyp ( *p* < 0.05).

**Conclusion** The result of our study showed that CMC/ACP cement-based (5% CAG and 10% CAG) demonstrated amorphous characteristic, which can stabilize calcium ions and phosphate group (ACP). In addition, the modification of gypsum using CMC/ACP as cement-based extended the time of setting.

## Introduction

The concept of minimal invasive is done by removing infected dentin and maintaining affected dentin. Infected dentin is the outermost layer of caries containing bacteria and their byproducts, demineralized inorganic components, denaturation of collagen fibrils, and loss of vital odontoblast. 1 2 3 Meanwhile, on the affected dentin layer, denaturation has occurred, but it is bacteria free, has intact collagen, and still allows remineralization. 1 2 4 In the natural remineralization of dentin, there are two interrelated components, namely, collagen, which acts as a scaffold, and noncollagen protein, namely, dentin matrix protein 1 (DMP1), which plays an important role as a regulator and stabilizer of the nucleation of apatite minerals. 5 6 DMP1 has a high affinity for calcium ions and can bind to collagen through electrostatic interactions. DMP1 triggers the formation of nanocluster amorphous calcium phosphate (ACP) precursors. Then, DMP1 and ACP will form the DMP1/ACP nanocomplex. DMP1 works to stabilize ACP so that it does not aggregate and remains in nano size. Furthermore, the DMP1/ACP nanocomplex will penetrate the gap zone between collagens and form hydroxyapatite crystals so that intrafibrillar remineralization occurs. 7 In the caries process, some DMP1s will experience degradation of functions so that remineralization does not occur optimally on the demineralized dentin surface. 5

In the dentine remineralization process with a biomimetic strategy, the material used is expected to replace the function of degraded components such as DMP1. Several alternative materials can replace the function of DMP1 as a noncollagen protein that have been studied and used to stabilize ACP, namely, polyacrylic acid and polyaspartic acid, phosphorylated chitosan, and carboxymethyl chitosan (CMC). 5 Several past studies had looked for natural materials that may have an effect on the prevention of dentin demineralization. 5 8 9 One of the compounds obtained from natural materials is CMC, a derivative of chitosan and it is easily obtained from the crustacean shell. 10 CMC is rich in carboxyl groups, so it has a high calcium affinity in the CMC/ACP complex, which plays a role in the remineralization process. 4 Recent research by Chen et al and Annisa et al confirms that CMC/ACP in scaffold preparation can replace the role of DMP1 in the remineralization process in the formation of hydroxyapatite both extrafibrillar and intrafibrillar. 5 11

CMC/ACP preparations in scaffold form have been characterized. Several studies have shown that the material triggers guided tissue remineralization in both dentin and enamel, but the scaffold form was lacking good handling properties and is not practical in dentistry due to the short duration of use, complex storage, and complicated processing. 5 11 12 13 According to Yamin et al and Maharti et al, a mixture of gypsum and CMC/ACP can be one of the solutions to overcome this problem, and their study showed that evidently this mixture yields positive effects in dentin remineralization. 14 15 To date, none of the research explains how the preparation of CMC/ACP in the form of powder can be mixed as a cement material suitable for the field of dentistry as dentin remineralization. The current study aims to characterize the CMC/ACP material as a cement-based mixture.

## Materials and Methods

### Preparation of CMC/ACP Powder Materials

Preparation for CMC/ACP powder: 0.555 g of CaCl _2_ was added to 10 mL deionized water under stirring for 5 minutes, and then 0.498 g of K _2_ HPO _4_ was added to this solution under stirring at 500 rpm. Then 2.5 g of CMC (PUI Kitosan and Material Maju, Universitas Sumatra Utara, Medan, Indonesia) was added to 20 mL of deionized water under stirring at 1,000 rpm until the CMC powder completely dissolved. Next, the CMC solution was added dropwise to the mixed solution (CaCl _2_ + K _2_ HPO _4_ ) to form a CMC/ACP gel. The gel was immediately frozen at −80°C for 2 hours and then lyophilized in a vacuum freeze dryer intermittent time (at 12, 4, and 8 hours) to form a dry material of CMC/ACP that could be crushed into a powder using a mortar.

### Preparation of CMC/ACP Post-Milled Powder

The powder resulting from lyophilization was subjected to a ball milling process using a high-energy shaker mill (Nano Center Indonesia, Serpong, Indonesia). The ball milling process was performed on the CMC/ACP powder for 30 minutes at 700 rpm with balls made of ceramic to obtain fine particle in the material. The above-mentioned milling time was chosen after a pilot study revealed that it yields the smallest particle smaller particles.

### Preparation of Cement-Based CMC/ACP

Cement-based CMC/ACP was prepared according to previous report with some modification. 5 14 15 16 17 CMC/ACP cement was prepared with gypsum (calcium sulfate hemihydrate/CaSO _4_ ∙½H _2_ O—Sigma Aldrich), CMC/ACP post-milled powder (CAPM), and water at a ratio of 2.5 g:0.25 g (10% ratio) and/or 0.125 g (5% ratio):1.9 mL, respectively. There were three experimental cement-based groups containing gypsum—calcium sulfate hemihydrate (Gyp), CMC/ACP gypsum cement 5% (5% CAG), and CMC/ACP gypsum cement 10% (10% CAG).

### Characterization of CMC/ACP, CAPM 5% CAG and 10% CAG

#### Fourier-Transform Infrared Spectroscopy Analysis

The functional groups present in the CMC, CMC/ACP, and CAPM, 5% CAG and 10% CAG were analyzed in the spectral range of 4,000 to 510 cm ^−1^ using Nicolet iS50 Fourier-Transform Infrared Spectrometer (Thermo Fisher Scientific, United States).

#### X-ray Diffraction Analysis

The X-ray diffraction (XRD) pattern of CMC, CAPM, 5% CAG and 10% CAG were recorded using an X-ray diffractometer (D8 DIS- COVER, Bruker, United States) with Cu K _α_ radiation (k = 1.54 degrees) and scanning rate of 1 step/s with step size of 0.1 degree/step. XRD test was performed to evaluate the crystallization phase of the material.

### Morphological Analysis

Morphological analysis was used to observe crystalline structures of all groups (Gyp, 5% CAG, and 10% CAG) by field emission scanning electron microscopy (SEM, FEI Quanta 400, The Netherlands).

### Setting Time

Samples of dimension 6 mm × 3 mm were prepared in three groups ( *n* = 6) consisting of Gyp, 5% CAG, and 10% CAG. A Vicat needle with 3N in weight and a flat-ended cylindrical needle tip of 1 mm in diameter were applied to the surface of the sample and indentations interval were 60 seconds. Setting time was recorded from the start of mixing to 60 seconds when the needle failed to penetrate a depth of more than 1 mm on the surface of the sample.

### Statistical Analysis

Statistical testing was performed using Windows, version 23 of IBM SPSS software. The degree of significance was set to *p-* value < 0.05.

## Results

### Characterization of CMC/ACP, CAPM, 5% CAG and 10% CAG

**Fig. 1:**
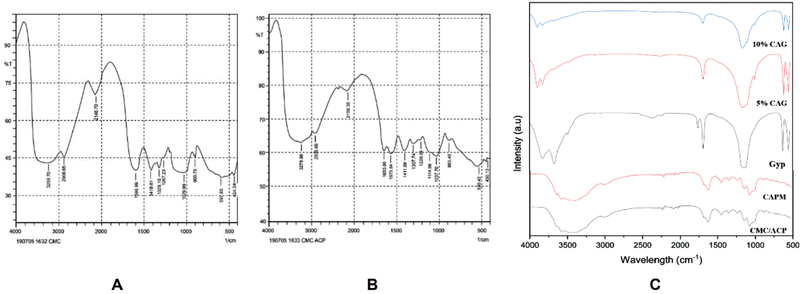
FTIR spectrum of ( **A** ) CMC, ( **B** ) CMC/ACP, ( **C** ) CMC/ACP, CAPM, Gyp, 5% CAG, and 10% CAG comparison.

Fourier-transform infrared spectroscopy (FTIR) spectra of CMC and CMC/ACP powder are shown in Fig. 1a, b . A band at 1,419.61 cm ^−1^ of the -CH2COOH and peaks of the -NH2 at 1,599.99 for CMC indicate the presence of CMC in the form of carboxymethyl (-COO-1) and amine (-NH2). Phosphate groups (PO4 ^3−^ ) were detected in the CMC/ACP material at 1,005.90 and 1,085.43 cm ^−1^ indicating interaction between Ca ^2+^ and CMC. Phosphate groups indicating calcium phosphate in the form of amorphous (ACP) were present in CMC/ACP. The milling process did not alter the functional groups of the CAPM compared with CMC/ACP ( Fig. 1c ). FTIR spectra of sulfate were evident in Gyp (1,121.28 cm ^−1^ ), 5% CAG (1,124.38 cm ^−1^ ), and 10% CAG (1,122.87 cm ^−1^ ), and carboxyl functional groups were detected at 1,681.3 to 3,549.31 cm ^−1^ (5% CAG) and 1,679.62 to 3,609.15 cm ^−1^ (10% CAG); 5% CAG and 10% CAG showed two peaks shifted to 1,606.63 and 1,415.68 cm ^−1^ indicating interaction of calcium ions and CMC. Additional phosphate groups at 1118.37 cm ^−1^ (5% CAG) and 1,085.43 cm ^−1^ (10% CAG) indicate that the amorphous forms were present in the cement-based CMC/ACP.

**Fig. 2:**
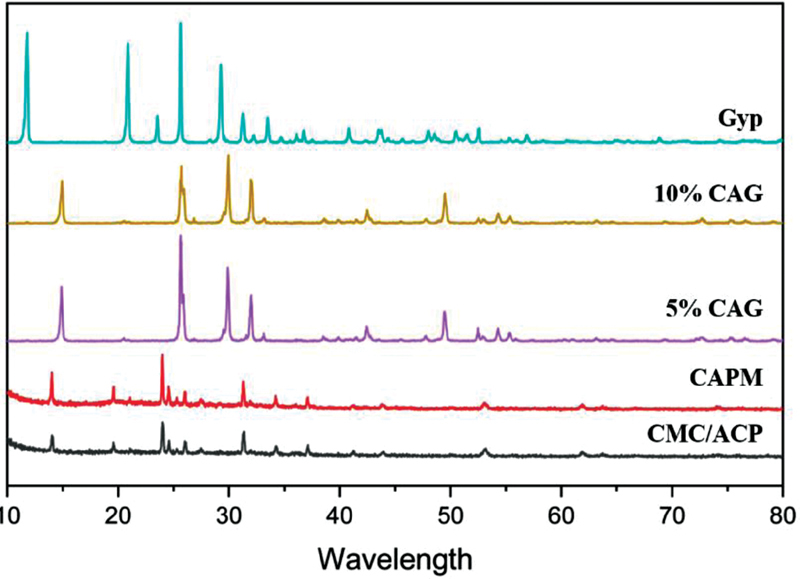
XRD spectrum of CMC/ACP, CAPM, 5% CAG, 10% CAG, and Gyp. The graphic showed no difference in diffraction spectra among the Gyp, 5% CAG, and 10% CAG groups. ACP, amorphous calcium phosphate; CAPM, CMC/ACP post-milled powder; CMC, carboxymethyl chitosan; Gyp, gypsum; XRD, X-ray diffraction.

XRD was used to identify the crystallization phase in the CMC/ACP processed by cryodesiccation and CAPM. The crystallization phase of calcium and dicalcium phosphate dihydrate was evident in CMC/ACP and CAPM. Fig. 2 shows that there is no difference in diffraction spectra among the Gyp, 5% CAG, and 10% CAG groups. The Gyp group showed higher crystal phase of nitratine, gypsum, and bassanite compared with cement-based CMC/ACP (5% CAG and 10% CAG).

### Morphology

**Fig. 3:**
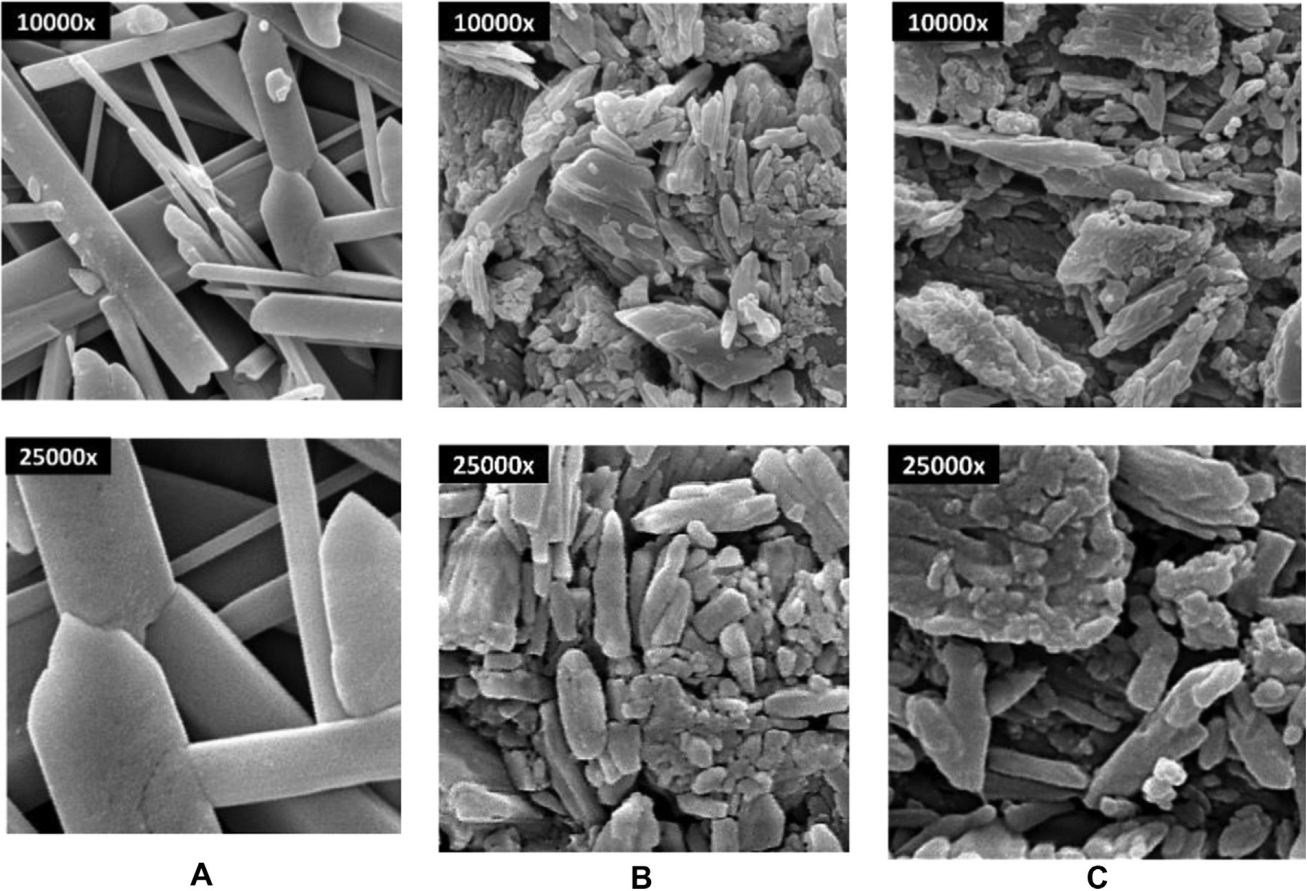
Morphology of ( **A** ) Gyp, ( **B** ) 5% CAG, ( **C** ) 10% CAG at 10.000× and 25.000 × . SEM images revealed that the CMC/ACP cement groups (5% CAG and 10% CAG) showed CMC/ACP cluster filled with hollow spaces between the gypsum crystals and aggregations surrounding the gypsum crystals were evident in the 5% CAG and 10% CAG cement groups. The CMC/ACP showed envelopes and attached to the crystalline structures of the gypsum. ACP, amorphous calcium phosphate; CAPM, CMC/ACP post-milled powder; CMC, carboxymethyl chitosan; Gyp, gypsum; SEM, scanning electron microscopy.

The morphology images of Gyp, 5% CAG, and 10% CAG were shown in Fig. 3a to c . Needle-shaped crystal and intermingled crystalline structures of gypsum were observed in Gyp. The porous structure between crystalline structures was evident in gypsum. The presence of CMC/ACP that filled hollow spaces between the gypsum crystals and the aggregations surrounding the gypsum crystals was evident in the 5% CAG and 10% CAG cement groups. The CMC/ACP showed envelopes and attached to the crystalline structures of the gypsum.

### Setting Time

**Fig. 4:**
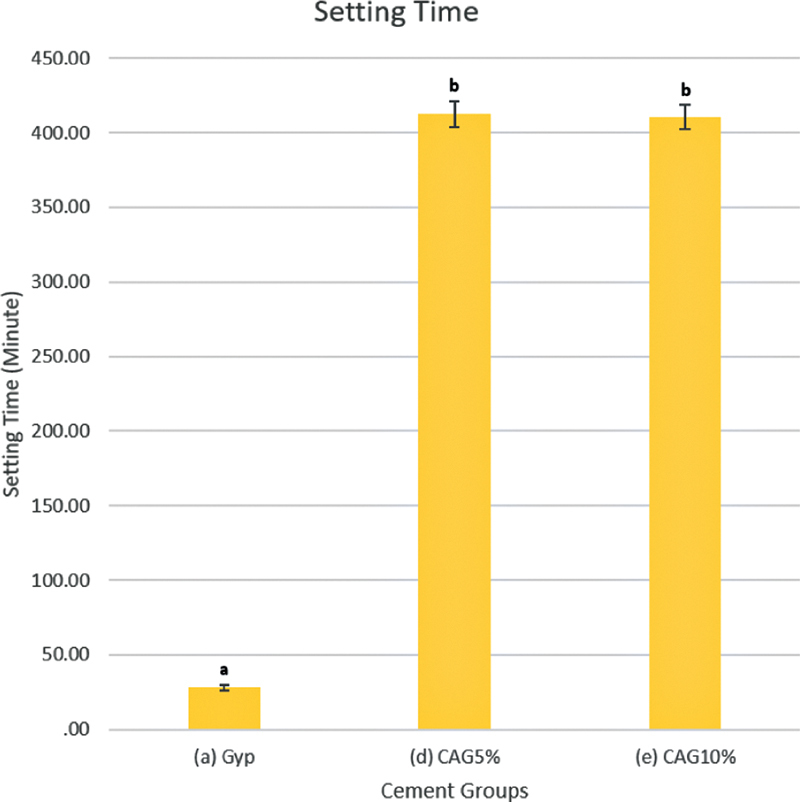
Setting time of Gyp, 5% CAG, and 10% CAG. The error bars represent the standard deviation. Bars with different letters are statistically significant ( *n* = 6; *p* < 0.05, Tukey's honestly significant difference). Gyp, gypsum.

The setting times of the materials are listed in Fig. 4 . The setting times of cement-based CMC/ACP in all groups (5% CAG and 10% CAG) ranged from 389.37 to 424.44 minutes, and the setting times of gypsum (Gyp) ranged from 25.62 to 30.88 minutes. Our findings further showed that the setting time of cement-based CMC/ACP (5% CAG and 10% CAG) in all groups was significantly longer than Gyp ( *p* < 0.05). This result indicated that setting time was altered in 5% CAG and 10% CAG compared with Gyp.

## Discussion

Chitosan is a natural polymer derived from the exoskeleton of crustaceans and one of the most abundant carbohydrates in nature. 10 18 Chitosan also has desirable characteristics such as biorenewability, biodegradability, biocompatibility, bioadhesivity, antimicrobial activity, nontoxicity, low immunogenicity, low cost, and accessibility. 10 18 The attempt to modify chitosan as a novel material or addition in restoration materials showed potential benefit in restorative dentistry. 19 20 21 22 However, chitosan has several limitations, such as highly ordered crystalline structure arising from solid hydrogen bonds and poor solubility in water. 10 18 23 The limitation of poor solubility of chitosan will limit its effectiveness in various processes, including modification as a cement-based material, but this can be overcome by depolymerization into CMC, which can lead to its preparation as a water-soluble derivatives. 10 18 23 ACP has been shown to be a material that plays a primary role in biomineralization. 24 25 The ACP and its modification could decrease demineralization and provide a potential method for caries management. 5 14 15 24 26 27 28 29 Stabilization of ACP by analogs of noncollagen protein has been studied in bottom-up remineralization strategy. 5 14 24 25 CMC as one of the natural analogs has a proven ability to stabilize ACP and prevent the particles from undergoing crystal transformation. 5 11 14 The attempt to modify the CMC/ACP into a gel has been conducted by Chen et al using lyophilization or a desiccation process in vacuum freeze dryer and has also been confirmed by Annisa et al and Setiati et al. 5 11 13 However, the gel form of CMC/ACP was complex to apply in clinical settings due to lack of handling properties, short duration of use, complex storage, and complicated processing.

Modification of CMC/ACP into a powder form in this study showed that the characteristic of CMC to stabilize ACP was evident in CMC/ACP from the FTIR spectra. The result of Chen et al indicated that different enhanced times of cryodesiccation did not alter this characteristic of CMC/ACP compared with CMC/ACP in gel formed. 5 CMC/ACP was ground into fine particles and the materials were blended using ball milling for 30 minutes. In the pilot study, the ball milling process did not decrease the particle size linearly with increasing times, and the smallest particle was evident in 30 minutes of milling. This result probably indicated that the flexible structure of polymers of CMC enables it to adopt complexation with metal ions, thus allowing the material to withstand the milling process. 10 The interesting finding of this study was a ball milling time of 30 minutes did not alter the functional groups or the mineral phase of CAPM compared with CMC/ACP. The interaction of carboxyl groups, which act as stabilizers of ACP, might be the reason for the stability of CAPM after the ball milling process. 5 10 14 18

Gypsum or calcium sulfate has been used in dentistry because of its biocompatibility and its ability to act as a carrier for other biomaterials. 14 17 The addition of viscous polymer (i.e., carboxymethyl cellulose and hyaluronan) shows better handling characteristics and mechanical properties. 30 The mixture of CMC/ACP as concentrated solution and gypsum on dentin remineralization has been described by Yamin et al and Maharti et al. 14 15 In this study, the porous structure of gypsum was evidently filled with CMC/ACP cluster when the cement was mixed in all groups, and similar phenomenon has been described by Subhi et al using chitosan material. 17 The addition of CAPM did not affect the crystalline phase of calcium sulfate in this research and similar study from Ding showed that chitosan matrix did not affect the crystalline phase of calcium phosphate cement. 31 The evidence of phosphate groups from FTIR spectrum finding in 5% CAG and 10% CAG indicates the presence of amorphous phase is expected to support remineralization process and treatment of decalcification. 5 27 32 33 CMC/ACP cement-based groups (5% CAG and 10% CAG) induce crystallization phase in a form of gypsum, nitratine and bassanite but lacked characteristic diffraction of crystalline hydroxyapatite that may be indicating the ACP phase associated with CMC. 5 Subhi et al stated that chitosan matrix did not affect the crystallization of calcium sulfate. 17 The pattern of CMC/ACP adsorption in 5% CAG and 10% CAG was observed in the SEM images and compared with Gyp ( Fig. 3B, C ). These findings provide concerning evidence from the above statement and by previous research from Low et al. 16 In addition, CMC/ACP cement-based groups (5% CAG and 10% CAG) showed better handling properties when manipulated.

In this study, the setting times of cement-based CMC/ACP groups (5% CAG and 10% CAG) were significantly larger compared with the setting time of Gyp. The results indicate that crystallization was compromised due to Ca ^2+^ ion stabilization by carboxyl groups from CMC/ACP, resulting in increased setting time. The ratio of powder/water prior to mixing was adapted from Subhi et al. 17 However, compared with the results from the present study in which gypsum hemihydrate is used, the results of Subhi et al are different due to the use of gypsum dihydrate, which is transformed into a hemihydrate phase. The amorphous calcium sulfate phase of gypsum can precipitate from an aqueous solution via hemihydrate. 34 Increased time of setting due to the amorphous phase could be the reason the crystallization of material was compromised. Therefore, the CMC as a polymer can be a strong inhibitor of dihydrate formation, resulting retardation of crystal growth and increase of setting time. 35 The extended time for the material for setting in the clinical scenario might be favorable for dentine remineralization. Kim et al stated that prolonged application time using remineralization agents can promote remineralization of the early carious lesion. 36 The limitation of this study is that this study only assessed the characteristic and setting time of cement-based CMC/ACP (5% CAG and 10% CAG). Future research should include the physical properties, cell viability, and dentin remineralization in the tooth caries model.

## Conclusion

The result of our study showed that cement-based CMC/ACP (5% CAG and 10% CAG) demonstrated amorphous characteristics, which can stabilize calcium ions and phosphate group (ACP). In addition, the modification of gypsum using CMC/ACP as cement-based extended the time of setting.
